# Molecular Evolution and Characterization of Fish Stathmin Genes

**DOI:** 10.3390/ani10081328

**Published:** 2020-08-01

**Authors:** Jun Cao, Xiuzhu Cheng

**Affiliations:** School of Life Sciences, Jiangsu University, Zhenjiang 212013, China; 18351276523@163.com

**Keywords:** stathmin, molecular evolution, functional divergence, neurotoxicity

## Abstract

**Simple Summary:**

Stathmin is a highly conserved microtubule remodeling protein. Here, 175 putative stathmin genes were identified in 27 species of fish. Gene organization, motif distribution, divergence of duplicated genes, functional divergence, synteny relationship, and protein-protein interaction were performed to investigate their evolutionary history. In addition, expression profiles of some stathmins were examined under dimethoate treatment. The results will provide useful references for further functional analyses.

**Abstract:**

Stathmin is a highly conserved microtubule remodeling protein, involved in many biological processes such as signal transduction, cell proliferation, neurogenesis and so on. However, little evolutional information has been reported about this gene family in fish. In this study, 175 stathmin genes were identified in 27 species of fish. Conserved exon-intron structure and motif distributions were found in each group. Divergence of duplicated genes implied the species’ adaptation to the environment. Functional divergence suggested that the evolution of stathmin is mainly influenced by purifying selection, and some residues may undergo positive selection. Moreover, synteny relationship near the stathmin locus was relatively conserved in some fish. Network analyses also exhibited 74 interactions, implying functional diversity. The expression pattern of some stathmin genes was also investigated under pesticide stress. These will provide useful references for their functional research in the future.

## 1. Introduction

Microtubules are a kind of hollow polymer formed by the polymerization of α and β tubulin into protofilaments, which constitute a part of the cytoskeleton, and provide structure and shape for cells [1]. Many biological processes such as cell proliferation, signal transduction, material transport, cell cycle progression and movement are associated with microtubules [2]. Stathmin is a phosphorylated protein of microtubule remodeling, which is involved in a variety of signal pathways. Some studies have indicated that overexpression of stathmin is associated with a variety of malignant tumors, also known as oncoprotein 18 (Op18) [3,4]. At the molecular level, stathmin destroys the stability of microtubules by sequestering the tubulin heterodimers to reduce the available amount of tubulin, and by binding the exposed protofilaments to induce microtubule instability [5].

Stathmin family proteins consist of STMN1, STMN2 (SCG10), STMN3 (SCLIP), and STMN4 (RB3). They have a C-terminal α-helix structure (predicted stathmin-like domain) combined with tubulin heterodimers and an N-terminal phosphorylation sites’ regulatory region [6,7]. Moreover, their expression and tissue distribution are also different. STMN1 is mainly expressed in the cytosol, and the other three members are distributed on vesicle and Golgi membranes [8,9]. Stathmin activity can be regulated by phosphorylation, in which some kinases such as MAPK, PKA and CaMKII are involved [10]. Phosphorylation of some serine residues (Ser16, Ser25, Ser38 and Ser 63) in the N-terminal regulatory region facilitates the release of tubulin heterodimer and creates conditions for microtubule assembly [4,11]. In addition, some conserved cysteine residues are related to the location of Golgi apparatus [12].

Stathmins control cell mitosis and their abnormal expression can affect cell division. They are abundant in the nervous system and can induce the growth and branching of axons and regulate the differentiation and proliferation of neurons [13]. Increased evidences have indicated that stathmin family genes are associated with a variety of tumors [14,15]. Stathmin is highly expressed in tumor tissue, which is related to drug resistance and tumor metastasis [16,17,18]. Therefore, stathmin can be used as a biomarker and target for clinical diagnosis and treatment. T cells are involved in the formation of immune synapses, the transport of intracellular substances, and the directional secretion of cytokines. Relocation of the microtubule tissue center is the key to activating T cells. Deletion of stathmin hinders this process, thus affecting T-cell lysis [19,20]. It is suggested that stathmin activated T cells by regulating the microtubule network. In addition, knockout or overexpression of stmn4 can change the depolymerization activity of microtubules, and then affect neurogenesis in zebrafish [12].

As the first animal group with an adaptive and innate immune system, fish research can deepen our understanding of the immune system, and also contribute to the protection of food supply and environmental monitoring [21,22,23]. However, little evolutional information has been reported about this family gene in fish [12,24]. Here, 175 putative stathmins were identified in 27 species of fish. Next, phylogeny, exon-intron structure, motif distribution, divergence of duplicated genes, functional divergence, expression profiles, and protein–protein interaction were performed to investigate their evolutionary characteristics. It will have an important reference value for the functional research of this gene family in the future.

## 2. Materials and Methods

### 2.1. Identification of Putative Stathmin Genes in 27 Species of Fish

Based on the model species, economic value, and key evolutionary position, 27 species of fish, including Amazon molly (*Poecilia formosa*), Atlantic salmon (*Salmo salar*), Blue tilapia (*Oreochromis aureus*), Cave fish (*Astyanax mexicanus*), Climbing perch (*Anabas testudineus*), Cod (*Gadus morhua*), Coelacanth (*Latimeria chalumnae*), Electric eel (*Electrophorus electricus*), Elephant shark (*Callorhinchus milii*), Fugu (*Takifugu rubripes*), Guppy (*Poecilia reticulata*), Huchen (*Hucho hucho*), Jewelled blenny (*Salarias fasciatus*), Lamprey (*Petromyzon marinus*), Medaka (*Oryzias latipes*), Ocean sunfish (*Mola mola*), Pinecone soldierfish (*Myripristis murdjan*), Platyfish (*Xiphophorus maculatus*), Round goby (*Neogobius melanostomus*), Shortfin molly (*Poecilia mexicana*), Spotted gar (*Lepisosteus oculatus*), Stickleback (*Gasterosteus aculeatus*), Tetraodon (*Tetraodon nigroviridis*), Tilapia (*Oreochromis niloticus*), Tongue sole (*Cynoglossus semilaevis*), Western mosquitofish (*Gambusia affinis*), and Zebrafish (*Danio rerio*), were considered for this study. A BLAST (Basic Local Alignment Search Tool) search was carried out in the Ensembl database [25] based on the hidden Markov model (HMM) of stathmin-like domain (pfam00836) to identify potential stathmin proteins in the 27 species of fish. Next, Pfam [26] and CDD [27] databases were further used to verify their authenticity. Some candidates without this conserved domain will be not further considered. ProtParam [28] and CELLO [29] were used to predict their biochemical characteristics and subcellular distribution, respectively.

### 2.2. Phylogeny, Exon-Intron Structure, and Motif Analysis of the Stathmin Gene Family

To explore their phylogenetic relationships, a multiple sequence alignment of these amino acid sequences was first performed using the MUSCLE (Multiple sequence comparison by log-expectation) method [30]. Next, MEGA 6 [31] was used to construct a neighbor-joining (NJ) tree with a 1000 bootstrap and *p*-distance substitution model and a maximum likelihood (ML) tree with a 100 bootstrap and Jones-Taylor-Thornton (JTT) model, respectively. One stathmin gene (FBgn0266521) of a fruit fly (*Drosophila melanogaster*) was used as an outgroup for constructing these phylogenetic trees. In addition, a species tree was also constructed based on the 16s rDNA sequences with an outgroup of a fruit fly. Gene organization was referred from the annotation information of the Ensembl database [25]. In addition, MEME [32] was used to identify conserved motifs with 6 to 50 width and parameters for up to eight motifs.

### 2.3. Difference Analyses of Duplicated Stathmin Genes

ClustalW (codons) in MEGA 6 [31] was used to align the nucleotide sequences of duplicated genes. K-Estimator 6.0 [33] was used to calculate their *Ka* (synonymous rate) and *Ks* (non-synonymous rate) values. Substitution events of the amino acid sites between duplicated genes were evaluated using *Ka/Ks* values.

### 2.4. Functional Divergence Analyses

To estimate which residues were responsible for functional changes among these stathmin groups, type-I functional divergence was calculated using DIVERGE (A software to detect functional divergence between member genes of a protein family) [34]. It refers to the difference of amino acids between two members of the duplicated genes, which means that these residues have changed in functional restriction [34]. The coefficient is significantly higher than 0, indicating the change of specific amino acid sites after duplication. Functional divergence of key amino acid sites was predicted by posterior analysis.

### 2.5. Synteny Analysis in Stathmin Genes

Cave fish stathmin gene (Ame_stmn3, ENSAMXG00000017478.2) was used to search homologous copies in Genomicus v78 [35]. In addition, the homologous genes near the Ame_stmn3 loci were also compared and analyzed in some fish.

### 2.6. Network Assembly of Protein-Protein Interaction

STRING (Search tool for the retrival of interacting genes/proteins) database [36] was used to assemble the interaction networks. Some sources including gene fusion, co-expression, databases, neighborhood, text-mining, co-occurrence and experiments exist in it [36]. Stathmin genes of zebrafish were submitted to the STRING with the following parameter: 0.400 medium confidence; maximum 5 and 10 interactors on the first and the second shell, respectively.

### 2.7. Materials, Pesticide Exposure, RNA Sequencing and Analysis

All of the procedures have been approved by the animal care and use committee (ethical approval number: UJS-LAER-2018120901). Pyrethroid and carbamate pesticides have great toxicity to aquatic organisms. Therefore, dimethoate, an organophosphorus pesticide, was used in this study. Four species of fish (zebrafish, fugu, stickleback, and medaka) were obtained from aquaculture farms. After 2 days of adaptation at 22–23 °C, the fish in the experimental group were treated with dimethoate (2.1 mg/L) for 24 h [37]. Meanwhile, the fish in the mock group were still kept in fresh water. RNA samples of three biological replicates were collected together, and the same amount of RNA was taken for RNA-seq in OE Biotech (Shanghai). Expression level of UniGene is estimated by calculating the value of fragments per kilobase of transcripts per million fragments (FPKM) in each sample.

## 3. Results and Discussion

### 3.1. Identification and Phylogenetic Analysis of the Stathmin Gene Family in 27 Species of Fish

**Figure 1:**
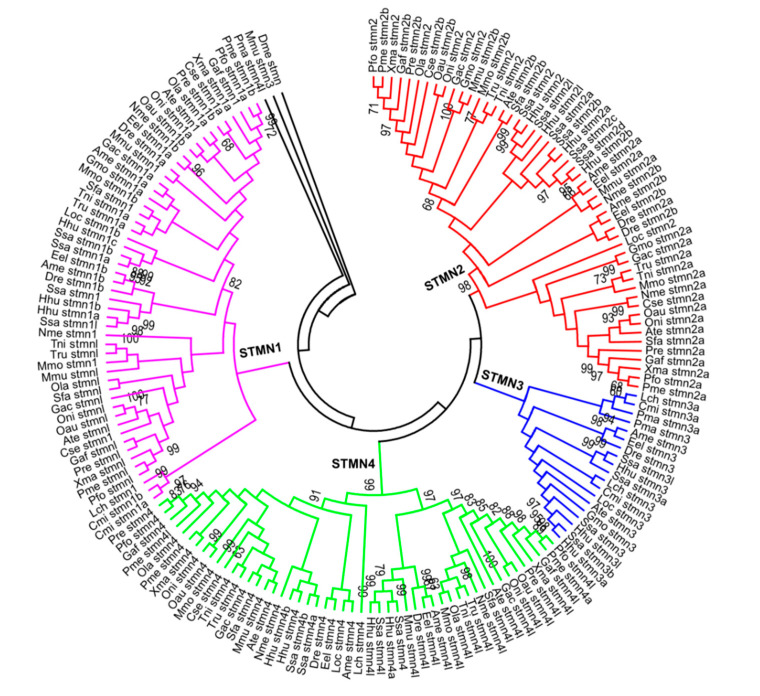
Phylogenetic relationships of the stathmin genes in 27 species of fish. A neighbor-joining (NJ) phylogenetic tree was constructed and four groups (STMN1, STMN2, STMN3, and STMN4) were classified based on the phylogeny and sequence similarity. Dme_stmn gene (FBgn0266521) of a fruit fly was used as an outgroup.

We first identified 175 putative stathmin genes from 27 species of fish (Figure S1), which encode 80–255 residues with pI values ranging from 4.87 to 9.89 (Table S1). All of these predicted stathmin proteins were significantly hydrophobic. Most of them were located in the cytoplasm or nucleus predicted by CELLO [29]. To elucidate the phylogenetic relationship of the stathmin family genes in the 27 species of fish, NJ and ML evolutionary trees were constructed. Notably, a very similar topology was found between them (Figure 1 and Figure S2). Here, the NJ tree was used for further analysis (Figure 1). Most of the stathmin members were divided into four groups (STMN1, STMN2, STMN3, and STMN4) based on the phylogeny and sequence similarity (Figure 1). This is consistent with the previous classification [6,7]. The evolutionary relationship of Mmu_stmn3 and Pma_stmn4l is far away from other members and is not clustered in any group. They will not be further analyzed in this study. STMN1 and STMN2 groups contain the maximum number of 52 members, representing over 29.7% of the total number, while the STMN3 group only contains 19 members. Moreover, we also found that some stathmin genes were distributed in tandem in the genomes of *S. salar*, *H. hucho*, and others, which indicated that tandem duplication accelerated the amplification of stathmin genes in these fish species (Table S1).

### 3.2. Gene Organization and Motif Distribution of the Stathmin Members

**Figure 2:**
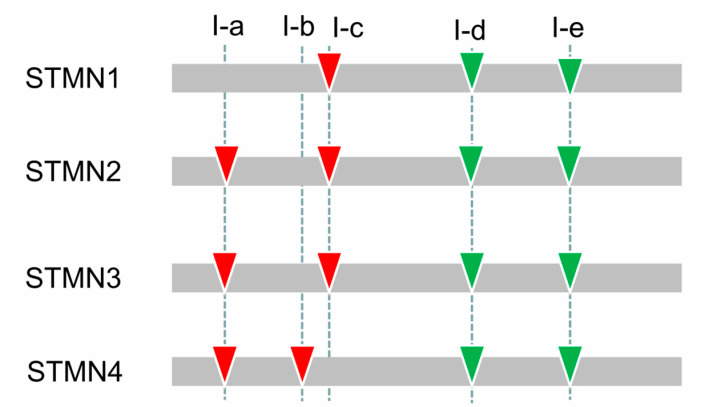
Exon–Intron organization of the stathmin genes in different groups. The inverted triangles with green and red marks indicate the insertion positions of phase 0 and 1 introns, respectively. Five conserved introns (I-a, I-b, I-c, I-d, and I-e) were also named and marked.

Intron gain and loss increase the complexity of gene structure in evolution [38,39]. To understand the structural diversity of stathmin genes, we compared their exon-intron structures (Figure 2). Five relatively conservative introns are shown in Figure 2, and are named I-a, I-b, I-c, I-d, and I-e, respectively. Phase distribution and insertion of intron I-d and I-e were also conserved in these four groups, suggesting their common origin. Some intron gain and loss events were also found in the evolution of stathmin genes. For instance, I-c intron was lost, but I-b intron was gained in the process of STMN4 evolution. STMN1 lost I-a intron, which was still present in other three groups (Figure 2). In addition, several non-conserved introns were also observed in the variable domain. One previous study has shown that when the position of intron changes, the extension or shortening of exon is the source of sequence variation [40]. Therefore, differences of gene structure increase the divergence of the family genes in the evolutionary process.

**Figure 3:**
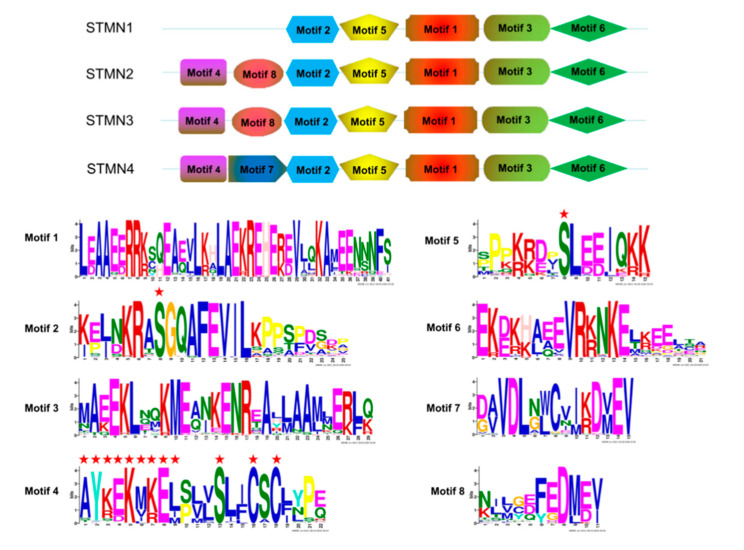
Different motif composition and distribution of the stathmin proteins. The height of a letter indicates its relative frequency at the given position for the amino acid. Eight conserved motifs are shown here. Some key residues are marked with asterisks.

Next, MEME [32] was used to compare their structural diversity. Eight conserved motifs were found among them (Figure 3). Generally speaking, most members of each group have the same motif, indicating their functional similarity [38,41]. In addition, some group-specific motifs were also found. For instance, motif 7 was specific for the STMN4 group. In the N-terminal extension, a consensus sequence (AYKEMKEL) and two conserved cysteine residues were found in motif 4, which could be palmitosylated to facilitate stathmin recruitment to Golgi. Mutations at these two sites significantly reduced stathmin’s ability to be recruited into Golgi apparatus [12]. Moreover, some conserved serine residues were also found in motif 2, motif 4, and motif 5. Phosphorylation of these serine sites reduces stathmin’s ability to depolymerize microtubules [4,11]. By converting these serine residues into alanine residues, stathmin’s depolymerization activity was enhanced [12]. Therefore, these specific regions or sites play important roles in the functional properties of stathmin members.

### 3.3. Divergence Analysis of Duplicated Stathmin Genes

**Figure 4:**
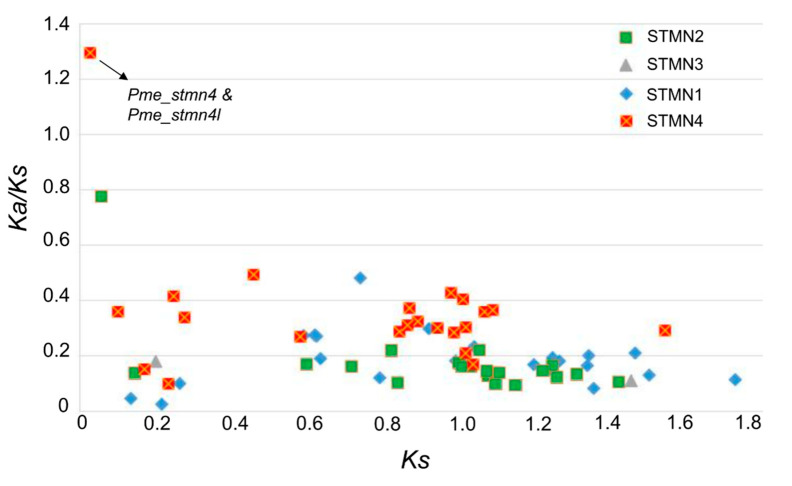
Relative rate of divergence for duplicated stathmin genes.

Gene duplication is an important factor in promoting the complexity of organisms. Duplicated genes usually undergo different evolutionary fates, such as pseudogenetization, subfunctionalization, and neofunctionalization [42]. In the process of evolution, duplicated genes face different selection pressures, which are represented by two parameters: *Ka* and *Ks*. To explore the divergence of duplicated stathmin genes, their selective pressure was investigated. A total of 23, 22, 3, and 22 pairs of duplicated stathmins were found in STMN1, STMN2, STMN3, and STMN4, respectively. The results indicated that most duplicated stathmins were subjected to purifying selection (Figure 4). Only one pair of genes (*Pme_stmn4* and *Pme_stmn4l*) had a *Ka/Ks* value above 1, indicating that they were under selective pressure. Sequence analysis indicated that, compared with the *Pme_stmn4* gene, one segment (motif 6) was inserted into the 3` end of the *Pme_stmn4l* gene, which led to further divergence between them. Although the function of motif 6 needs further study, these duplicated genes may provide new genetic material for organisms to cope with environmental changes.

### 3.4. Functional Divergence Analysis among Different Stathmin Groups

Amino acid substitution can lead to protein functional changes. To verify this view, we used DIVERGE [34] to evaluate the type-I functional divergence among different stathmin groups. Four groups of homologous stathmin proteins were compared, and the evolution rate of each amino acid was calculated. The results show that all coefficient values (θ) of functional divergence are less than 1 among these groups (Table 1). This indicates that most stathmin members have obvious site-specific selection restrictions, which affect the evolution of group-specific functions after diversification [34]. Moreover, according to the site-specific profiles and the cut-off value of posterior probability, the key residues leading to functional divergence are predicted. There were significant differences in the number and site distribution in each group pair. For instance, no key residues were predicted for STMN2/STMN3 group pairs regardless of the cut-off value of 0.5 or 0.7. The maximum theta value (0.867878) was found in STMN1/STMN4, indicating the selective relaxation of specific sites or a higher evolutionary rate between them. In addition, we also examined seven functional divergent sites identified in STMN3/STMN1 groups and found that four of them were located in motif 2, two and one in motif 5 and motif 1, respectively. Additionally, four sites were located near the conserved phosphorylated serine responsible for microtubule depolymerization. Therefore, the different evolutionary rate of some residues may lead to functional changes in evolution.

### 3.5. Synteny Analysis near the Stathmin Gene Loci

**Figure 5:**
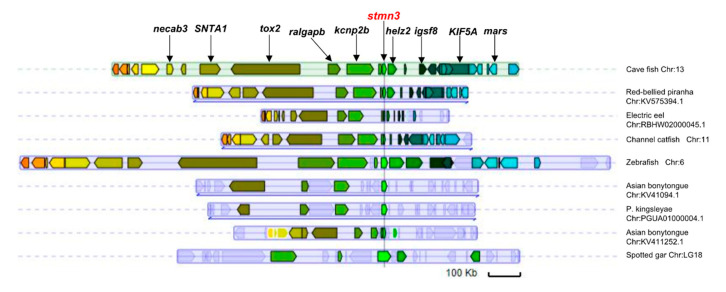
Synteny analyses of the chromosome loci harboring stmn3 genes in several fish. Some genes near Ame_stmn3 loci present conserved synteny in most of the identified species.

Synteny refers to the conservative evolutionary relationship between genome regions, which provides a framework for inferring the common ancestor of genes [43,44]. Thus, synteny analysis is an important tool to study gene function and evolution. The stmn3 gene of cave fish (Ame_stmn3, ENSAMXG00000017478.2) was used to carry out the synteny analysis in the Genomicus database v78 [35]. The results indicated that some loci near stmn3 showed conserved synteny in some fish (Figure 5). In addition, some gene deletions or insertions were also found in the neighboring regions of stmn3 in some fish. For example, compared with the cave fish, some genes (tox2, SNTA1, KIF5A, etc.) near stmn3 were lost in the spotted gar, suggesting a distant evolutionary relationship between them.

### 3.6. Interaction Network Analysis of the Stathmin Members

**Figure 6:**
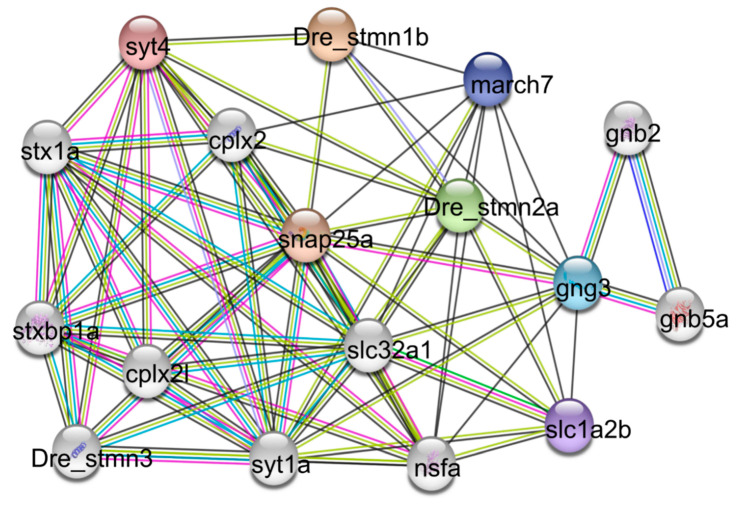
Functional network assembly of the zebrafish stathmin genes. Seventy-four interactions were exhibited among a total of 17 genes.

STRING database [36], based on prediction and experimental analysis, was used to construct a protein interaction network to explore which members might interact with stathmin. The network showed 74 interactions between three stathmins and 14 other genes (Figure 6). Some membrane-associated RING-CH protein 7 (march7), N-ethylmaleimide sensitive factor (nsf), synatotagmin (syt), syntaxin (stx), and 25 kDa synaptosome-associated protein (snap25) were predicted as the main interactors of stathmins. March7 belongs to a family of ubiquitin E3 ligase, which is involved in the neuronal development and T cell proliferation [45,46] Nsf is a necessary ATPase for membrane fusion. Zebrafish *nsf* mutants exhibited significant neuropeptide protein accumulation, synaptic transmission, and nerve activity disorder [47,48]. Therefore, nsf plays a key role in the nervous system. It promotes the proper fusion of synaptic vesicles and enables neurons to communicate through chemical synapses [49]. Synaptic transmission is a basic biological process to maintain neuronal communication. Syt is a major Ca^2+^ sensor that activates vesicular exocytosis and releases presynaptic neurotransmitters [50]. The release of glutamate from neurons in syt knock-out mice decreased significantly, suggesting that the release of presynaptic neurotransmitters plays an important function [51]. Stx regulates exocytosis, membrane fusion, and the docking of secretory particles on the plasma membrane [52]. Snap25 is a presynaptic membrane protein specifically expressed in neuron cells [53]. It is involved in many biological processes, such as neural signal transduction, axon growth, neurotransmitter release, etc. [54]. In this study, we found that these proteins were predicted to interact with stathmins (Figure 6). This suggests that stathmins may be involved in the process of neurotransmission and development. In addition, complexin-2 (cplx2), G-proteins (gnb2 and gng3), and solute carrier family proteins (slc1a2b and slc32a1) were also found in the network (Figure 6), suggesting that stathmins may play roles in a variety of physiological processes.

### 3.7. Expression Profiles of the Stathmin Genes under Dimethoate Stress

**Figure 7:**
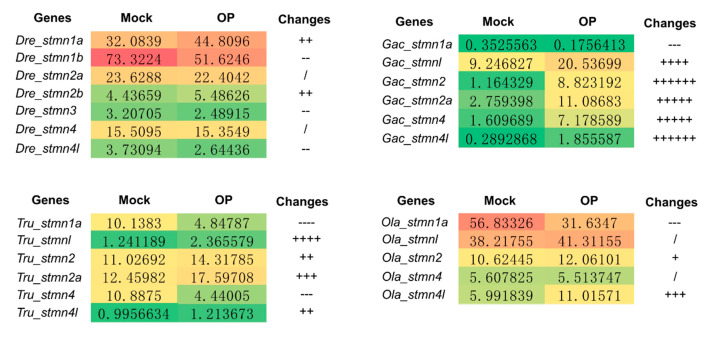
Expression profiles of the stathmin genes under dimethoate treatment in zebrafish, fugu, stickleback, and medaka.

Pesticides widely interfere with many physiological processes of aquatic organisms [55]. In this study, the expression profiles of stathmin genes under dimethoate stress were investigated by RNA-seq. The results indicated that compared with the mock group, 54.2% and 29.2% of stathmin genes were up-regulated and down-regulated under dimethoate stress, respectively (Figure 7). Among them, the expression level of *Gac_stmn2* and *Gac_stmn4l* genes were increased by 7.1 and 6.4 times, respectively, under this treatment. Previous studies have indicated that stathmins play an important role in the development and regeneration of the nervous system by mediating the growth and branching of axons [12,13,56]. Moreover, dieldrin, an organochlorine pesticide, can induce neurotoxicity in fish by altering the expression level of some proteins including stathmin 1a [57]. Here, our study also indicated that dimethoate changed the transcriptional level of most stathmin, implying potential neurotoxic effects on fish. In addition, the expression patterns of several duplicated stathmins, such as *Dre_stmn1a* and *Dre_stmn1b*; *Tru_stmn1a* and *Tru_stmnl*; *Gac_stmn4* and *Gac_stmn4l* and so on, were also investigated. The results indicated that their expression patterns were different under dimethoate stress (Figure 7). It suggests that these genes have different expression profiles after duplication, which is helpful for organisms to adapt to the changing environment in evolution.

## 4. Conclusions

A comparative analysis of the stathmin family was carried out in 27 species of fish. They were classified into four groups based on phylogenetic tree. The conserved exon–intron structure and motif distribution in each group implied functional relevance. Divergence of duplicated genes and some sites related to the functional divergence suggested an adaptation to the environment. Synteny analysis suggested relatively conserved evolution characteristics near the stathmin loci in some fish. In addition, 74 interactions were exhibited in the network analyses, implying functional diversity. Pesticides can cause environmental pollution and seriously affect the growth and development of aquatic organisms. The different expression patterns of stathmin genes under the dimethoate treatment indicated potential immune function and provided clues for further functional research. In addition, some stathmin genes with differentially expressed profiles under pesticide can also be used as a potential indicator in environmental monitoring. All provide useful references for further study.

## Figures and Tables

**Table 1:** Type I functional divergence estimated in fish stathmin paralogs.

Comparison	θ	SE	LRT	N (0.5)	N (0.7)
STMN2/STMN3	0.106549	0.110902	0.181231	0	0
STMN2/STMN1	0.440025	0.151336	20.19846	12	8
STMN2/STMN4	0.525401	0.15557	28.169269	18	10
STMN3/STMN1	0.548957	0.173591	14.761422	11	6
STMN3/STMN4	0.424753	0.152923	11.700322	19	6
STMN1/STMN4	0.867878	0.178251	56.703338	46	20

SE: standard error; LRT: likelihood ratio test; N: numbers of divergent residues.

## Supplementary Materials

The following are available online at https://www.mdpi.com/2076-2615/10/8/1328/s1, Table S1: Stathmin genes identified in this study, Figure S1: Phylogenetic tree of 27 species of fish based on 16S rDNA sequences. Fruitfly was used as an outgroup, Figure S2: Phylogenetic relationships of the stathmin genes in 27 species of fish. ML phylogenetic tree was constructed and four groups (STMN1, STMN2, STMN3, and STMN4) were classified here. Dme_stmn gene (FBgn0266521) of fruitfly was used as an outgroup.
